# Cockayne Syndrome Phenotype in a Sudanese Child With a Homozygous ERCC8 Splice-Site Variant of Uncertain Significance: A Case Report

**DOI:** 10.7759/cureus.117244

**Published:** 2026-09-30

**Authors:** Fatma Ragab, Alaa Alhag

**Affiliations:** 1 General Medicine, Independent Practice, Denver, USA; 2 Pediatrics and Child Health, Dr. Erfan & Bagedo General Hospital, Jeddah, SAU

**Keywords:** acquired microcephaly, cockayne syndrome, ercc8 gene, global developmental delay, photosensitivity, postnatal growth failure, progeroid facial features, progressive neurologic impairment, variant of uncertain significance, whole-exome sequencing

## Abstract

Cockayne syndrome (CS) is a rare autosomal recessive DNA repair disorder caused by biallelic pathogenic variants in excision repair cross-complementation group 6 (*ERCC6*) or group 8 (*ERCC8*) and characterized by progressive postnatal growth failure, neurodevelopmental impairment, photosensitivity, and premature-aging features. Its evolving multisystem phenotype can complicate early clinical recognition, particularly when molecular testing identifies a variant of uncertain significance (VUS).

We report a Sudanese girl residing in Saudi Arabia, born to consanguineous parents, with normal birth parameters and initially satisfactory development who, from late infancy, developed progressive postnatal growth failure, acquired microcephaly, and global developmental delay. Subsequent photosensitivity, progeroid features, spasticity, ataxia, tremor, and reported hearing and visual impairment raised clinical suspicion for CS, although Sanjad-Sakati syndrome was initially considered. Whole-exome sequencing (WES) identified a homozygous canonical acceptor splice-site variant in *ERCC8* (NM_000082.3:c.482-2A>T), predicted to disrupt normal splicing. Although listed as likely pathogenic in ClinVar, it was classified by the testing laboratory as a VUS. Given the strong phenotypic concordance, the variant was interpreted as supportive of, but not independently diagnostic for, CS.

This case underscores the importance of longitudinal phenotypic assessment for recognizing CS and highlights the need for cautious integration of molecular findings with the clinical phenotype when genetic testing identifies a VUS.

## Introduction

Cockayne syndrome (CS) is a rare autosomal recessive multisystem disorder characterized by progressive postnatal growth failure, microcephaly, neurodevelopmental impairment, sensory abnormalities, cutaneous photosensitivity, and a characteristic progeroid appearance [1]. Recent literature reports an incidence of approximately 2.7 per million births across Western Europe and Japan [2], consistent with earlier nationwide Japanese data estimating an incidence of 2.77 per million births and a prevalence of approximately one in 2.5 million [3]. The clinical spectrum is heterogeneous, ranging from severe congenital or early-onset disease to milder forms presenting later in childhood, and progressive neurologic manifestations may include developmental impairment, cerebellar ataxia, spasticity, peripheral neuropathy, and deterioration of vision and hearing [1].

CS is associated with pathogenic variants in excision repair cross-complementation group 6 (*ERCC6*) or group 8 (*ERCC8*), which encode the Cockayne syndrome group B (CSB) and Cockayne syndrome group A (CSA) proteins, respectively, and are involved in transcription-coupled nucleotide excision repair [4]. *ERCC8*-related CS accounts for a substantial minority of cases and may result from multiple types of genetic alterations, including missense, nonsense, frameshift, deletion, and splice-site variants [5,6]. Because of the broad phenotypic spectrum and overlap with other childhood neurodevelopmental disorders, diagnostic interpretation may be challenging, particularly when molecular testing identifies a variant of uncertain significance (VUS) [1].

We present a case of a Sudanese girl residing in Saudi Arabia, born to consanguineous parents, with normal birth parameters followed by progressive postnatal growth failure, acquired microcephaly, neurodevelopmental impairment, photosensitivity, sensory abnormalities, and progressive neurologic dysfunction. Whole-exome sequencing (WES) identified a homozygous canonical splice-site variant in* ERCC8 *that was listed as likely pathogenic in ClinVar [7] but classified by the testing laboratory as a VUS. This case illustrates the importance of integrating longitudinal clinical findings with cautious interpretation of genomic results in children with a phenotype strongly suggestive of CS.

## Case presentation

A Sudanese girl residing in Saudi Arabia was initially evaluated at approximately three years and six months of age for progressive postnatal growth failure, developmental delay, motor impairment, speech delay, and feeding and swallowing difficulties. She was born at term by uncomplicated vaginal delivery following an unremarkable pregnancy. Her birth weight was 2.8 kg, length was 49 cm, and head circumference was 34 cm. Her parents are first cousins, and there was no known family history of a similar disorder, unexplained childhood deaths, or neurologic disease. The family pedigree is shown in Figure 1.

**Figure 1:**
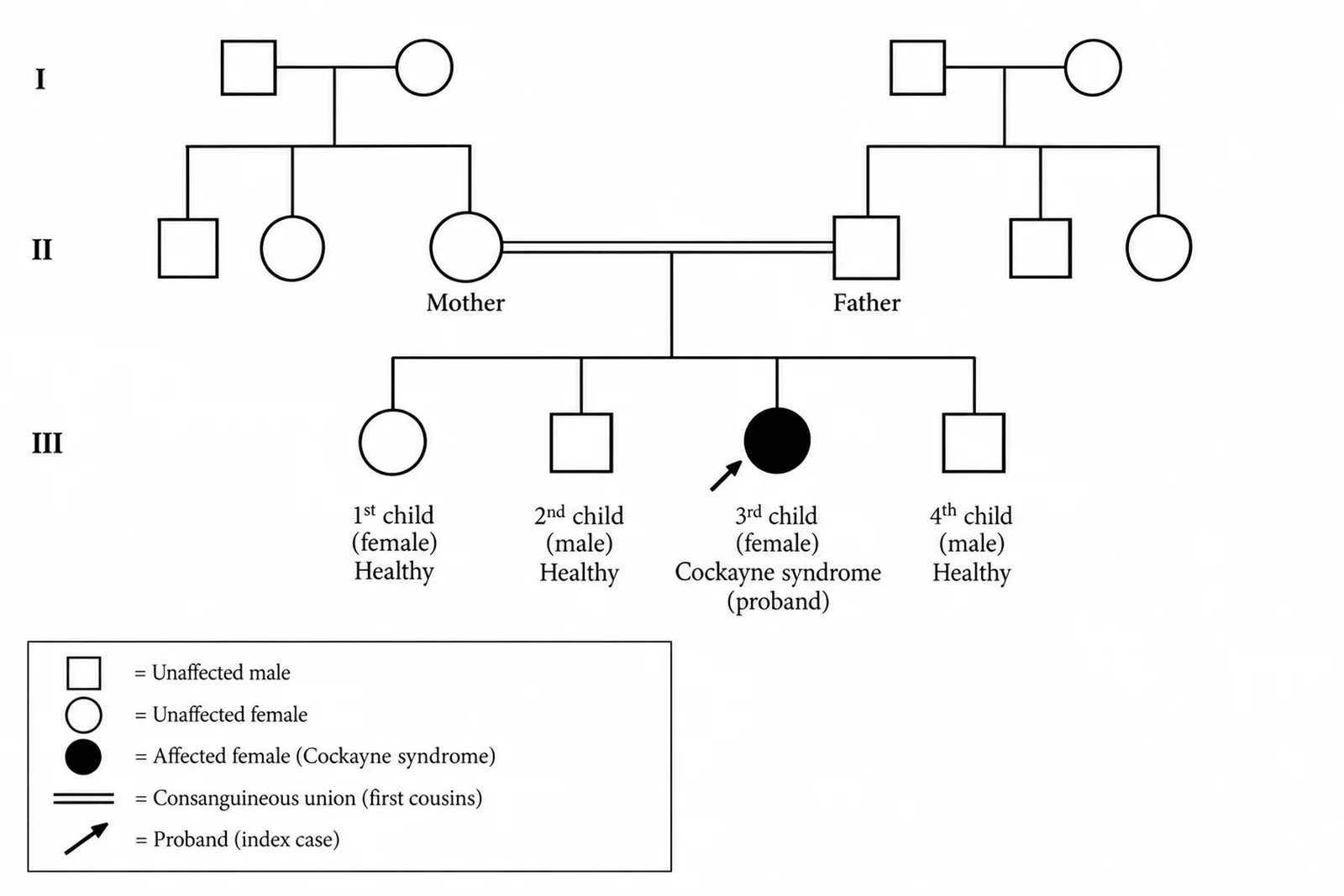
Family pedigree of the patient The proband (arrow) is the third child of consanguineous parents who are first cousins. The proband has a clinical phenotype strongly suggestive of Cockayne syndrome, while her three siblings and the other reported family members are unaffected.

Early growth and development were initially satisfactory. Beginning at approximately eight to 12 months of age, weight gain and linear growth progressively slowed. Head growth also decelerated, with acquired microcephaly becoming apparent during the second year of life. Gross motor milestones included head control at four months, rolling at six months, independent sitting at nine months, crawling at 12 months, and standing with support at approximately 18 months. Independent walking was never achieved. Fine motor abilities included reaching for and transferring objects and picking up small objects, although age-appropriate fine motor skills remained impaired. Expressive language development was markedly delayed; at the most recent assessment, she communicated primarily through gestures and a few single words while understanding some simple commands.

Feeding difficulties became increasingly apparent between two and three years of age. She had difficulty chewing solid foods and was predominantly given soft or mashed foods. Meals were prolonged, typically lasting approximately 30-45 minutes, and she occasionally coughed or choked while drinking liquids. No aspiration events were documented, and there was no history of nasogastric or gastrostomy feeding. Because of persistent poor weight gain, she received calorie-dense foods and oral nutritional supplementation.

At four years and 10 months of age, her weight was 7.5 kg, height was 85 cm, and head circumference was 47.5 cm. She had a thin, narrow face with sunken cheeks, prominent eyes, a thin, elongated nose, and loss of subcutaneous facial fat, producing a progeroid appearance. The skin was thin and dry, with increased pigmentation over sun-exposed areas in the setting of reported photosensitivity. Multiple dental caries were present. Neurologic examination demonstrated reduced muscle bulk, increased tone predominantly in the lower limbs, brisk deep tendon reflexes, impaired coordination, ataxia, and poor balance. She could sit independently and stand only with support. During supported ambulation, her gait was broad-based and unsteady. At the most recent follow-up, the family reported marked tremor during attempts at supported ambulation, contributing to worsening gait instability and recurrent falls. No significant resting tremor was observed on examination. There was no definite history of seizures or loss of consciousness. Mild limitation of ankle range of motion was present, although no major fixed contractures were identified. Her clinical appearance is shown in Figure 2.

**Figure 2:**
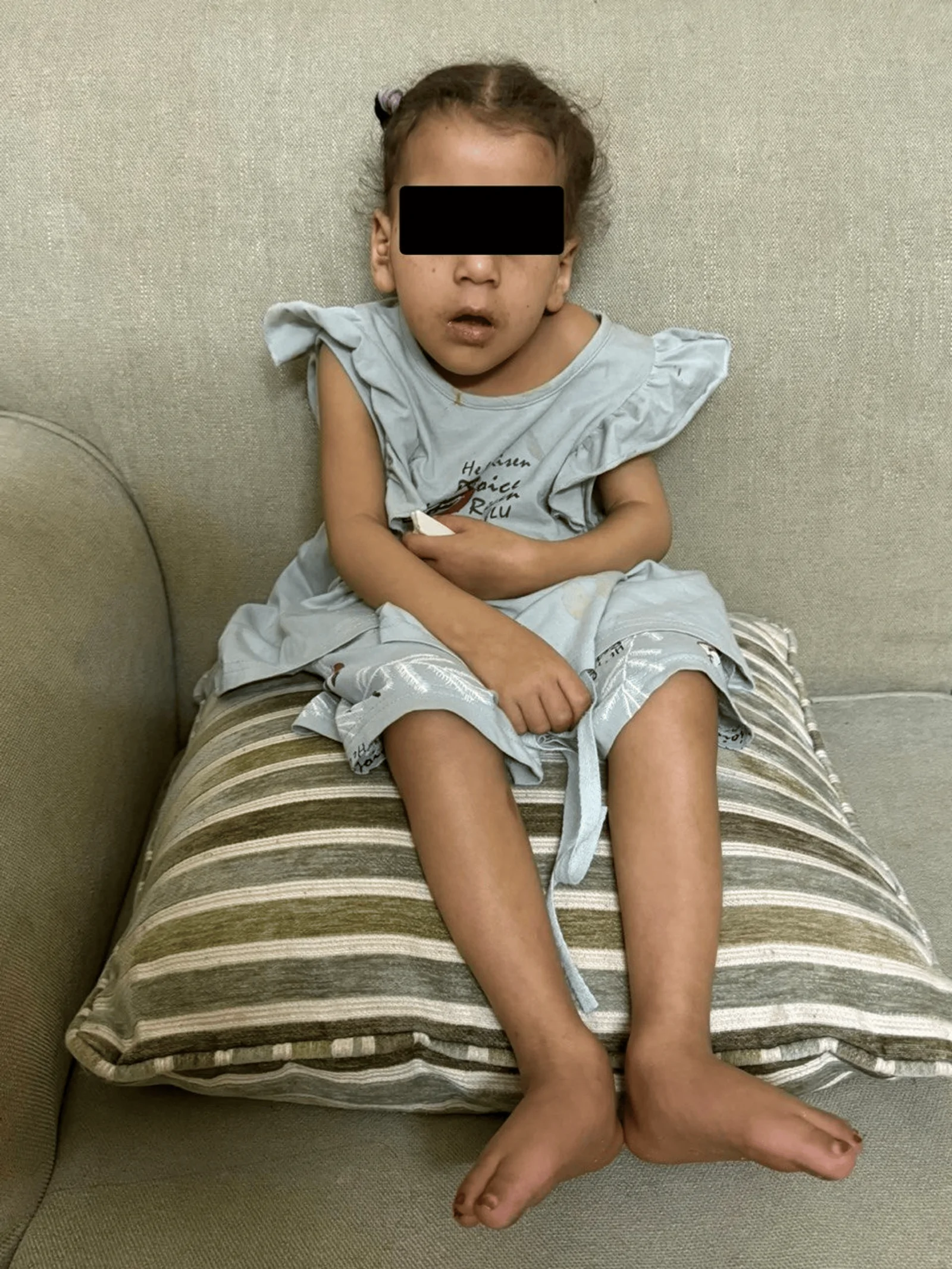
Clinical appearance of the patient at four years and 10 months Full-body photograph demonstrating a slender habitus and reduced subcutaneous tissue. Written informed consent to include the image in an open-access article was obtained from the patient's parent.

A closer view of the characteristic facial features is shown in Figure 3.

**Figure 3:**
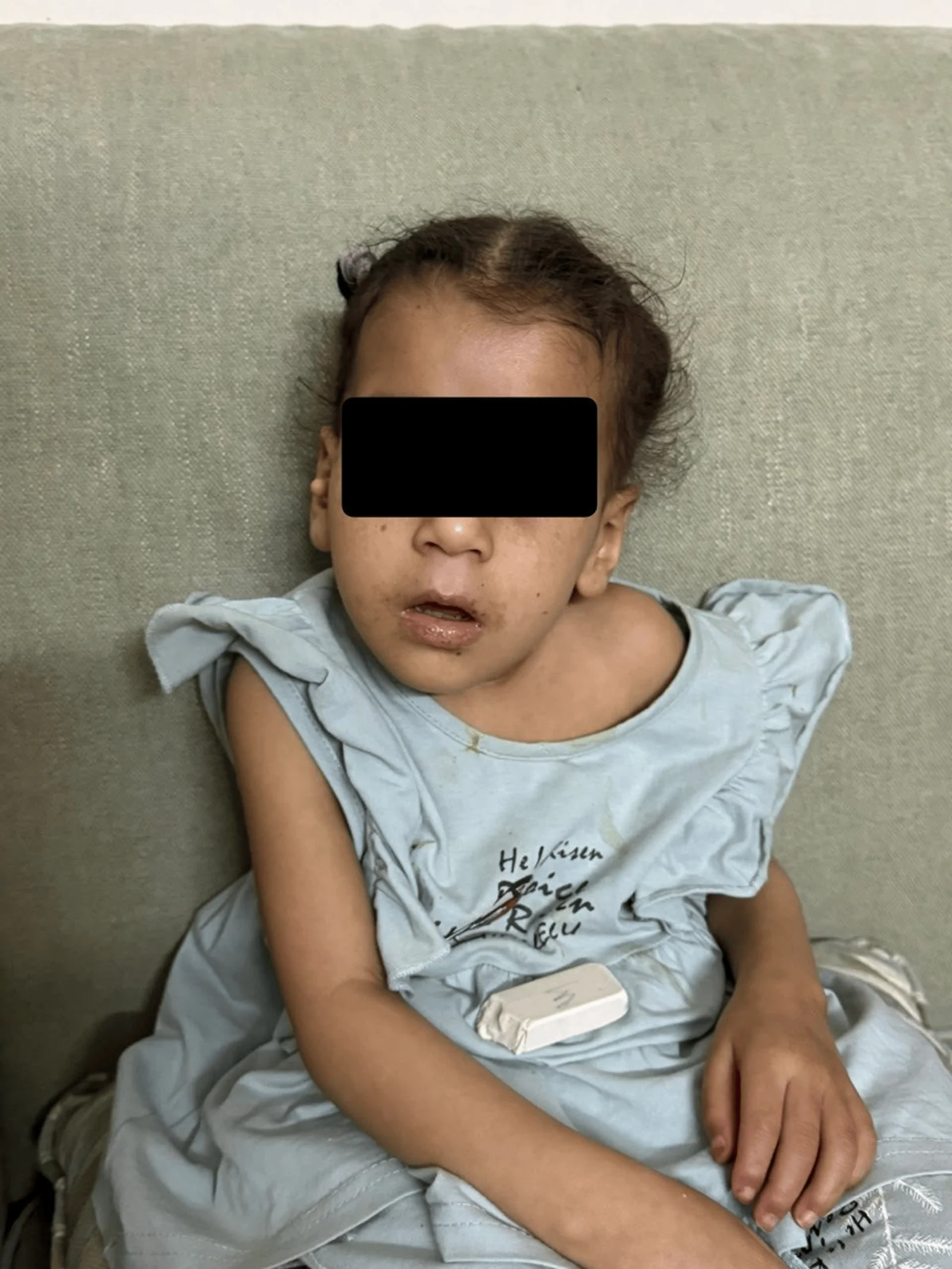
Characteristic facial features of the patient Close-up facial photograph demonstrating a thin, narrow face, sunken cheeks, and reduced subcutaneous facial fat. Written informed consent to include the image in an open-access article was obtained from the patient's parent.

Additional manifestations included reported hearing and visual impairment. She required assistance with feeding, dressing, toileting, and mobility. Despite her developmental limitations, she maintained eye contact, recognized her parents, smiled responsively, and interacted with familiar caregivers.

Because the initial presentation was dominated by severe growth restriction and developmental delay, Sanjad-Sakati syndrome was initially considered. As the clinical course evolved, however, the combination of progressive postnatal growth failure, acquired microcephaly, characteristic facial appearance, photosensitivity, progressive neurologic impairment, sensory abnormalities, and feeding difficulties increased clinical suspicion for CS.

WES subsequently identified a homozygous *ERCC8* variant, NM_000082.3:c.482-2A>T, affecting the canonical acceptor splice site. The testing laboratory classified the variant as a VUS and predicted disruption of normal splicing with skipping of the in-frame exon. The laboratory report also noted that ClinVar listed the variant as likely pathogenic at the time of reporting; however, under the laboratory’s implementation of American College of Medical Genetics and Genomics (ACMG)/Association for Molecular Pathology (AMP)/Clinical Genome Resource (ClinGen) criteria [8,9], it remained classified as a VUS. No additional clinically relevant variants explaining the phenotype were identified. Because the molecular finding alone was not considered diagnostic, it was interpreted in the context of the patient’s strongly concordant clinical phenotype. Parental segregation testing and future reanalysis of the sequence data were recommended.

Management has remained supportive and multidisciplinary. She receives nutritional supplementation, physiotherapy, speech and feeding therapy, and ongoing pediatric, neurologic, ophthalmologic, audiologic, dental, and genetic follow-up. Genetic counseling regarding autosomal recessive inheritance was provided to the family. At the most recent follow-up, she remained clinically stable without recent hospitalization but continued to demonstrate severe growth restriction, acquired microcephaly, global developmental delay, progressive motor dysfunction, feeding difficulties, and dependence on caregivers for most activities of daily living.

## Discussion

CS is a clinically heterogeneous progressive multisystem disorder, and recognition can be difficult during infancy because many of its characteristic manifestations develop over time [1]. The clinical course in our patient closely resembles the classic CS type I phenotype. She had normal prenatal growth and apparently satisfactory early development, followed during the first year of life by progressive postnatal growth failure, acquired microcephaly, developmental delay, and worsening neurologic dysfunction. The subsequent appearance of photosensitivity, a characteristic cachectic-progeroid facial appearance, reported hearing and visual impairment, dental caries, feeding difficulties, spasticity, ataxia, tremor, and progressive impairment of mobility further strengthened the clinical suspicion of CS [1,10].

The patient's phenotype is particularly consistent with the recognized spectrum of* ERCC8*-related disease.* ERCC8* encodes the CSA protein, which participates in transcription-coupled nucleotide excision repair. Pathogenic variants in *ERCC8* account for a substantial minority of CS cases and include missense, nonsense, frameshift, deletion, and splice-altering variants [1,5,11,12]. Although considerable clinical variability exists across the CS phenotypic spectrum [11,13],* ERCC8*-associated disease is frequently reported in patients with the classic type I phenotype [5,10]. A recent series of eight children with *ERCC8*-related CS found type I disease to be the most common subtype and described manifestations including microcephaly, growth impairment, developmental delay, motor abnormalities, spasticity, ataxia, dental disease, photosensitivity, and sensory involvement [10].

An important feature of the present case is the homozygous *ERCC8* canonical acceptor splice-site variant NM_000082.3:c.482-2A>T identified by WES. The testing laboratory predicted disruption of normal splicing but classified the variant as a VUS. This distinction is clinically important. Current diagnostic guidance indicates that identification of biallelic VUSs does not by itself establish or exclude a molecular diagnosis of CS [1]. Therefore, the molecular result in this patient should not be interpreted in isolation, despite the striking concordance between her phenotype and* ERCC8*-related CS.

The potential biological relevance of a splice-site alteration is nevertheless supported by previously reported *ERCC8 *variants affecting RNA processing. Chebly et al. described a homozygous *ERCC8* donor splice-site variant, c.843+1G>C, in a patient with CS and demonstrated abnormal incorporation of intronic sequence through RNA analysis [11]. Similarly, Schalk et al. demonstrated that a deep intronic *ERCC8 *variant caused insertion of a cryptic exon and validated the abnormal splicing effect using transcript analysis and a minigene assay [13]. More recent *ERCC8* reports have also identified splice-site defects among pathogenic variants associated with CS [10]. These observations provide biological plausibility for a splice-altering mechanism in our patient but do not establish pathogenicity of the specific c.482-2A>T variant.

The progressive nature of the disorder is also illustrated by the patient's evolving motor manifestations. She never achieved independent walking and developed lower-limb spasticity, impaired balance, and ataxia. More recently, marked tremor during supported ambulation has contributed to worsening instability and recurrent falls. Progressive central and peripheral neurologic dysfunction is characteristic of CS, and tremor, spasticity, ataxia, abnormal gait, and inability to walk have all been described within the clinical spectrum [14]. The evolution of these findings demonstrates the importance of longitudinal assessment, because the diagnostic phenotype may become considerably more recognizable as the child ages [1].

Management of CS remains primarily supportive and requires multidisciplinary care [1,10,15]. Nutritional and feeding assessment, physical therapy, management of spasticity and contractures, ophthalmologic and audiologic surveillance, dental care, sun protection, and continued neurologic monitoring are important components of care [1]. In our patient, nutritional supplementation, physiotherapy, speech and feeding therapy, ophthalmologic and audiologic follow-up, dental care, and genetic counseling have formed the basis of management. Recent data in *ERCC8*-related CS suggest that systematic rehabilitation may reduce the severity of musculoskeletal complications such as Achilles tendon contractures, reinforcing the importance of maintaining physical therapy as neurologic disability progresses [10].

Limitations

This report has several limitations. Functional RNA studies were not performed to confirm the predicted effect of the *ERCC8* splice-site variant on mRNA splicing, and parental segregation testing was not available. Therefore, although the patient’s phenotype is highly consistent with CS, the identified variant remains classified as a VUS and should not be considered independently diagnostic. The broad molecular heterogeneity of CS and the diversity of reported *ERCC6 *and *ERCC8 *variants further underscore the need for cautious interpretation of individual molecular findings [16]. Future RNA studies, segregation analysis, and periodic reanalysis of genomic data may provide evidence relevant to its eventual reclassification.

## Conclusions

CS should be considered in children with normal birth parameters who develop progressive postnatal growth failure, acquired microcephaly, developmental impairment, photosensitivity, sensory abnormalities, and neurologic decline. In this patient, the evolving phenotype was strongly suggestive of CS, although the homozygous canonical splice-site variant in ERCC8 remained classified as a VUS. Genomic findings, particularly VUSs, should therefore be interpreted in the context of the complete longitudinal phenotype rather than as isolated diagnostic evidence. Functional studies, segregation analysis, and periodic variant reanalysis may help clarify the significance of this variant. Early recognition and multidisciplinary supportive care remain essential for managing the neurologic, nutritional, sensory, and functional complications of this disorder.
